# Fracture risk in HIV and the need for guidelines: the Probono-1 Trial

**DOI:** 10.1186/1758-2652-13-S4-P99

**Published:** 2010-11-08

**Authors:** B Peters, H Isohanni, S Tillet, F Ibrahim, G Hampson, FMK Williams, MEO Perry, A Duncan, A Wierzbicki

**Affiliations:** 1 Kings College London, Harrison Wing, St Thomas' Hospital, London, UK; 2 Kings College London, Rheumatology, London, UK; 3 Kings College London, Chemical Pathology, London, UK; 4 Kings College London, Twin Research Unit, St Thomas' Hospital, London, UK

## Purpose of study

Fragility fractures are a common and increasing cause of morbidity and mortality in the general population; risk factors for fractures are commoner in HIV . This study aims to determine the prevalence and associations of low bone mineral density (BMD) and high fracture risk (FR) in an HIV cohort, suggest screening and management guidelines.

## Methods

A cross-sectional study of 223 randomly selected HIV-infected outpatients was undertaken. Recruitment was stratified by gender for age groups 30-39, 40-49 yrs and ≥50 yrs. Osteopenia and osteoporosis were diagnosed according to the WHO criteria. Patients completed a detailed questionnaire including combination HIV drugs (cART) history, & a dual-energy X-ray absorptiometry (DEXA) of Lumbar spine & Left Hip. Investigations included serum Ca, phosphate, 25OH vit D, alkaline phosphatase (Alk P) PTH, albumin, sex hormone binding globulin (SHBG), testosterone, CD4, HIV RNA. BMD risk factors were recorded including previous fractures, smoking, malabsorption, alcohol consumption, BMI, chronic diseases, physical activity index and past medication. FRAX score (10yr probability major fractures), and remaining lifetime fracture probability (RLFP) were calculated. Controls were from the Twin Research Unit at Kings College London. Data were analysed using multivariate logistic regression.

## Results

Demographics: 133(60%) were male, 106(48%) were Caucasian, 71(33%) had AIDS at diagnosis. 190(85%) were taking cART, of whom 50(26%) were on their first line therapy. Osteoporosis/osteopenia were present in 13%/39% of males, 11%/29% females, and was approximately 2.4/3.0 fold greater than age-matched controls. The overall mean 10 yr fracture risk was 3.16%. RLFP exceeded 1.0 in 76% HIV patients, and <20% controls.

Factors associated with low BMD after multivariate analysis: adjusted OR(95 % CI)/p-value BMI 0.90(0.83,0.96), p<0.001, Alk P 1.01(1.00,1.02), p 0.05, testosterone 1.04(1.01,1.07), p 0.01, Initiated cART 3.61(1.38,9.42), p 0.01. The lack of association with age is notable, adjusted OR 1.08(0.92,1.26), p 0.35

## Conclusions

Reduced bone mineral density and subsequent fracture risk is much commoner in patients with HIV compared to controls, especially, and those taking cART, and occurred across the age ranges. Hence screening for BMD and risk factors for fragility fractures is indicated in patients with HIV at a younger age than in the general population, especially if they are on cART.

